# Crow Deaths Caused by West Nile Virus during Winter

**DOI:** 10.3201/eid1312.070413

**Published:** 2007-12

**Authors:** Jennifer R. Dawson, Ward B. Stone, Gregory D. Ebel, David S. Young, David S. Galinski, Jason P. Pensabene, Mary A. Franke, Millicent Eidson, Laura D. Kramer

**Affiliations:** * New York State Department of Health, Albany, New York, USA; † New York State Department of Environmental Conservation, Delmar, New York, USA; ‡ New York State Department of Health, Slingerlands, New York, USA; § State University of New York, Albany, New York, USA; 1 Current affiliation: University of New Mexico, Albuquerque, New Mexico, USA

**Keywords:** West Nile virus, American Crow, lateral transmission, winter, roost, dispatch

## Abstract

In New York, an epizootic of American crow (*Corvus brachyrhynchos*) deaths from West Nile virus (WNV) infection occurred during winter 2004–2005, a cold season when mosquitoes are not active. Detection of WNV in feces collected at the roost suggests lateral transmission through contact or fecal contamination.

In the northern United States, West Nile virus (WNV) is thought to overwinter in hibernating mosquitoes (*1*). Because reports of birds dying of WNV infection during the winter are rare, we investigated the cause of crow deaths in New York during the winter of 2004–2005.

## The Study

Dead crows from a roost were reported to the Dutchess County Department of Health in December 2004 (Figure). The roost was located in coniferous and deciduous trees at the east end of the Mid-Hudson Bridge, Poughkeepsie, New York, USA. Because winter surveillance in Poughkeepsie had not previously confirmed WNV, the crows were not collected for testing.

**Figure:**
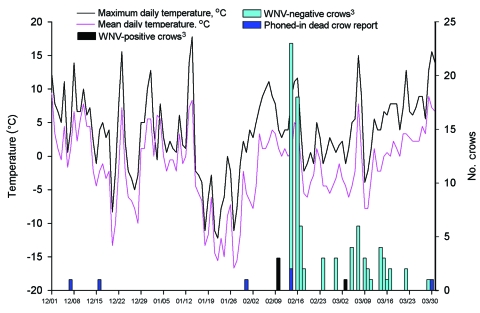
Crow deaths associated with West Nile virus (WNV) infection and maximum and mean temperatures for Poughkeepsie, New York, USA (December 1, 2004–March 31, 2005). Roost area was checked for crow carcasses at least every 48 hours after February 10, 2005. Temperature data were obtained from National Oceanic and Atmospheric Administration, Silver Spring, Maryland, USA. All 98 crow carcasses were tested for WNV by reverse transcription–PCR (RT-PCR) (*2*), VecTest, and Rapid Analyte Measurement Platform (*3*,*4*). Twelve were positive by all 3 tests; 1 crow collected on March 7, 2005, was positive by RT-PCR only.

However, after the third dead crow in January was reported, ground surveillance of the roost was initiated (Figure). Thereafter, carcasses were collected 4–5 times per week at a radius of 1/4 mile around the roost and were transported for necropsy to the New York State Department of Environmental Conservation. On March 1, 2005, the roost, culverts, and areas under the bridge were examined for overwintering mosquitoes. Temperature data from December 1, 2004, to March 31, 2005, were obtained from the National Oceanic and Atmospheric Administration, Silver Spring, Maryland, USA.

Oral swabs were collected from carcasses and screened by using VecTest (Medical Analysis Systems, Freemont, CA, USA) and Rapid Analyte Measurement Platform (RAMP; Response Biomedical Corp, Burnaby, British Columbia, Canada) (*3**,**4*). Brain tissue was submitted to the New York State Department of Health (NYSDoH) for testing by TaqMan reverse transcription–PCR (RT-PCR) and standard RT-PCR (*2**,**5*). When possible, blood clots were collected from heart chambers for antibody testing by ELISA (*6*). Ectoparasites were collected from some carcasses before necropsy and tested for WNV by TaqMan RT-PCR (*2*).

To characterize this WNV genotype, RNA was extracted from the homogenate of a WNV-positive crow kidney (strain 05000918) by using RNeasy (QIAGEN, Valencia, CA, USA). The envelope coding region was amplified in 3 overlapping fragments by using QIAGEN One-Step RT-PCR core kit. DNA was sequenced at the Wadsworth Center Molecular Genetics Core facility by using ABI 3100 or 3700 automated sequencers (Applied Biosystems, Foster City, CA, USA). We generated the sequence (GenBank accession no. DQ823132) by using the SeqMan module within Lasergene (DNASTAR, Madison, WI, USA) and compared it with previously characterized North American strains by using MegAlign within Lasergene.

We collected 45 fecal specimens from 12 sampling points in the roost and 10 from beneath 2 carcasses. Specimens were tested for WNV RNA by using TaqMan and standard RT-PCR (*2*) with minor modifications; 100 mg of each specimen was diluted in 1.0 mL of BA-1, homogenized, centrifuged, and sterile filtered. RNA was extracted from the filtrate by using RNeasy (QIAGEN), and RT-PCR was conducted.

From February 10 to March 29, 98 carcasses were collected from the roost area; of these, 12 (12.2%) were WNV-positive according to VecTest and RAMP and 13 (13.3%) were positive according to TaqMan RT-PCR (Figure). The crow isolate was characterized as the WN02 genotype (*7*).

Necropsy and histopathologic findings on WNV-positive crows (n = 13) were consistent with previously reported pathologic findings (*8*). Necropsy findings included low body weight (84.6%), enlarged spleen (23.1%), and enlarged liver (30.8%); histopathologic findings included slight to moderate encephalitis with mild, diffuse gliosis and occasional small foci of necrosis in the gray matter of the brain. Meningoencephalitis, characteristic of WNV-positive birds (*8*), was not observed. WNV-negative crows (n = 85) died from traumatic injuries (51.8%), predation (16.5%), avian pox (14.1%), pneumonia (11.8%), and poisoning (5.9%). Two pools of >20 lice (*Philopterus* spp.: *Mallophaga*) from 6 WNV-positive birds and 1 pool from 1 WNV-negative bird were tested; 6 positive pools were detected from 4 positive birds.

All 56 blood clots collected were seronegative by ELISA for flavivirus antibodies. Of the 45 fecal samples, 3 were WNV-positive; 2 of these (1 collected from beneath a WNV-positive crow; 1 from a random roost sampling point) had >800 pfu/mL, according to extrapolation from TaqMan RT-PCR.

No mosquito hibernacula were located in the areas examined, and no mosquito activity was observed by field workers. Maximum daily temperatures were >10°C for 6 days in December, 4 days in January and February, and 5 days in March; mean temperatures were <10°C throughout the epizootic (Figure).

## Conclusions

How WNV crow infections occurred during winter in New York when mosquito activity would have been limited is unclear (Figure). Reporting of crow carcasses can be as low as 10%; therefore, additional carcasses may have been observed and not reported before ground surveillance began (*9*). Initial crow infections could have occurred in November, when mean monthly temperature was >10°C and mosquito infection was more probable. Maximum daily temperatures >10°C occurred sporadically from December through March. However, mean temperatures remained at <10°C (Figure) and photoperiods at <12 h/day. Laboratory studies of wild-captured *Culex pipiens* L. females, the primary WNV vector in the northeastern United States, have shown that *Cx. pipiens* are unlikely to terminate diapause with photoperiods of <12 h/day and temperatures <10°C (*10*). Field studies in New York have shown that *Cx. pipiens* remain in overwintering locations until mid-April, at which time photoperiods are >12 h/day and mean temperatures >10°C (C. Drummond, NYSDoH Arbovirus Laboratories, unpub. data).

These winter deaths suggest a pattern of crow-to-crow transmission. WNV has been detected in blood–feather pulp of crows (*3*), and WNV-positive lice (*Philopterus spp.*) were collected from 4 WNV-positive crows. Research is needed on the risk for bird-to-bird viral transmission posed by ectoparasites, particularly to roost mates and nestlings. Scavenging of infected birds as a risk factor is supported by laboratory studies demonstrating WNV infection in crows after they ingested infected house sparrows (*Passer domesticus*) (*11*) and by chronic WNV infection in house sparrows and other bird species (*12*). Chronic infection in crows is unlikely given that laboratory studies have demonstrated 100% mortality rates within 5 days of infection (*11*).

Crow-to-crow transmission of WNV is supported by laboratory findings of fecal-shed WNV and contact transmission (*11**,**13*) and by WNV-positive results from oral and cloacal swabs used in VecTest and RAMP (*3**,**4*). In laboratory studies, crows shed WNV fecal titers as high as 10^8.8^ pfu/g (*13*). Our study provides the first evidence of fecal-shed WNV in the wild. In Illinois, healthy and WNV-infected crows roosted communally in summer (*14*); however, no additional evidence linked viremic crows and subsequent crow infections. Further study is needed on the role of summer and winter roosts and feces in the WNV transmission cycle. No human cases are known to be related to exposure to crow feces, although avoiding feces and wearing gloves when handling live or dead birds are recommended.

The role of birds in arbovirus overwintering and dissemination during migration has been suggested but is poorly understood. The last WNV-positive crow in this study was collected on March 29 as the roost was dispersing. Additional crows could have been infected before migrating to home territories. Radio-marked crows infected with WNV have traveled up to 4 km per night during the 5 days before they died (*14*). Thus, infected birds could transport the virus to new areas with active mosquitoes and contribute to the beginning of the WNV transmission cycle. We recommend additional study of winter WNV activity in crows and other bird species to determine their potential roles in arbovirus overwintering and the initiation of transmission when mosquitoes become active.
